# Evaluating the quality of indigenous chicken eggs in Gena Bossa district of Dawro zone, South Ethiopia

**DOI:** 10.1016/j.heliyon.2022.e12598

**Published:** 2022-12-23

**Authors:** Matawork Milkias, Meseret Molla

**Affiliations:** a Mizan-Tepi University College of Agriculture and Natura Resource Department of Animal Science, Ethiopia; b University of Gonder College of Veterinary Medicine and Animal Science, P. O. BOX 196, Ethiopia

**Keywords:** Agro-ecology, Egg quality, Education level, Gena Bossa, Ethiopia

## Abstract

This study was carried out in the Gena Bossa district to evaluate the quality of indigenous chicken eggs in different agro-ecology and education levels of chicken producers. A total of 108 fresh eggs were collected from randomly selected 36 households for the determination of egg quality parameters and three eggs from each household, 36 eggs from each Agro-ecology, and 27 eggs from each education level of farmers collected. Eggs collected from highland agro-ecologies had the highest egg weight (43.94 ± 0.42g), shell thickness (0.54 ± 0.01mm), albumen weight (23.31 ± 0.40g), and shell weight (5.25 ± 0.73g). Lowl and eggs had the highest yolk color score (8.65 ± 0.14). Eggs collected from primary second cycle educated farmers had the highest egg weight (44.41 ± 0.48g), albumen height (5.17 ± 0.17mm), Haugh unit score (75.34 ± 1.41%), yolk height (15.58 ± 0.33), albumen weight (23.65 ± 0.46g), and shell weight (5.33 ± 0.08g). Except for shell thickness (0.54 ± 0.11mm) all egg quality parameters were lowest for eggs collected from illiterate farmers. Egg weight was most appropriate in primary first cycle and primary second cycle interactions with respective highland and midland agro-ecologies. However, shell strength was most appropriate in the interactions of highland agro-ecology with illiterate education level. The combinations of primary second cycle with highland agro-ecology was most appropriate than other interactions for albumen weight. Depending on this conclusion, it will be better to investigate further studies on factors affecting internal and external qualities of the eggs in the study area.

## 1 Introduction

In developing African nations, farming is the base for financial advancement. Ethiopia is a developing African country and agriculture overwhelms the Ethiopian economy and contributes 45% of net household items (GDP) and gives more than 80% of work. Ethiopia has the most noteworthy animals populace in Africa and have 65, 40, 51, 8 and 49 million of cattle, sheep, goats, camels, and chickens, respectively (CSA, 2020a; Management Entity, 2021). World Bank (2017) report demonstrates that the animals’ division contributed almost 40% of rural Net Household Item (GDP), 20% of add up to GDP of the complete nation, and 20% of national remote trade profit (Matawork et al., 2018; NABC, 2010; PIF, 2010; Tsegaye, 2014).

The indigenous chickens of Ethiopia are nondescript breeds related to jungle fowl that differ in color, comb type, body conformation, weight, and whether or not they have shank feathers. The term "broodiness" (maternal instinct) is used (Solomon, 2008). They exhibit low output performance, late maturity, and slow development. According to estimates, native chickens lay 60 tiny, thick-shelled eggs per year with a rich yellow yolk. In some cases, the length of the egg-laying season and the number of eggs laid per cycle are longer in urban than in rural areas (Bosenu and Takele, 2014).

Poultry is the world's most popular livestock species, accounting for more than 30% of all animal protein needs (Kebede et al., 2017). Chicken's contribution to global protein production is expected to increase to 40% by 2050, with the importance of chicken expanding in developing countries (Matawork, 2018). In Ethiopia, where human food production is comparably fast, initial capital investment is modest, and uses are frequently made utilizing available family labor, chicken production is a mainstay of animal agriculture. Traditional, low-input methods and upgraded production systems using relatively modern technology, on the contrary, are clearly distinguished in the sector. More than 90% of the national poultry, egg, and meat output come from local poultry under the antiquated technique of chick rearing (Chaimiso 2018). Chicken has a tremendous socioeconomic impact in Ethiopia, affecting food security, money generation, and religious and other uses (Yizengaw et al., 2022; Gulilat et al., 2021).

Chicken eggs are well-known, nutritious, cost-effective, and straightforward to plan nourishment, as they give balanced sources of supplements for people of all ages (Matt et al., 2009). The egg is short-lived nourishment, due to the low efficiency of its characteristic security obstruction; when eggs are put away for a long period, particularly in unacceptable conditions, their initial quality goes harmed, getting to be, indeed, not great for utilization and require fast cooling and fridge medications amid capacity for keeping great quality (Bufano, 2000).

Egg quality refers to a set of all characteristics that impact the use of eggs as food products (Schwaegele, 2003). External factors such as cleanliness, freshness, egg weight, and shell quality are important to the acceptability of peeled eggs by consumers, among the many quality attributes (Sonaiya et al., 2004.). The internal quality of the eggs begins to deteriorate as soon as they are laid by the hens, in contrast to the exterior quality. While factors such as hen management and feeding can influence internal egg quality, egg handling and storage practices have a significant impact on the quality of eggs that reach consumers. There are many eggs produced in the study area, but so far producers were getting fewer quality eggs from their chickens. As a result, this study was designed with the goal of evaluating indigenous chicken egg quality in various agro-ecologies and educational levels of farmers in the SNNPR's Gena Bossa district.

## 2 Materials and methods

### 2.1 Description of the study area

**Figure 1:**
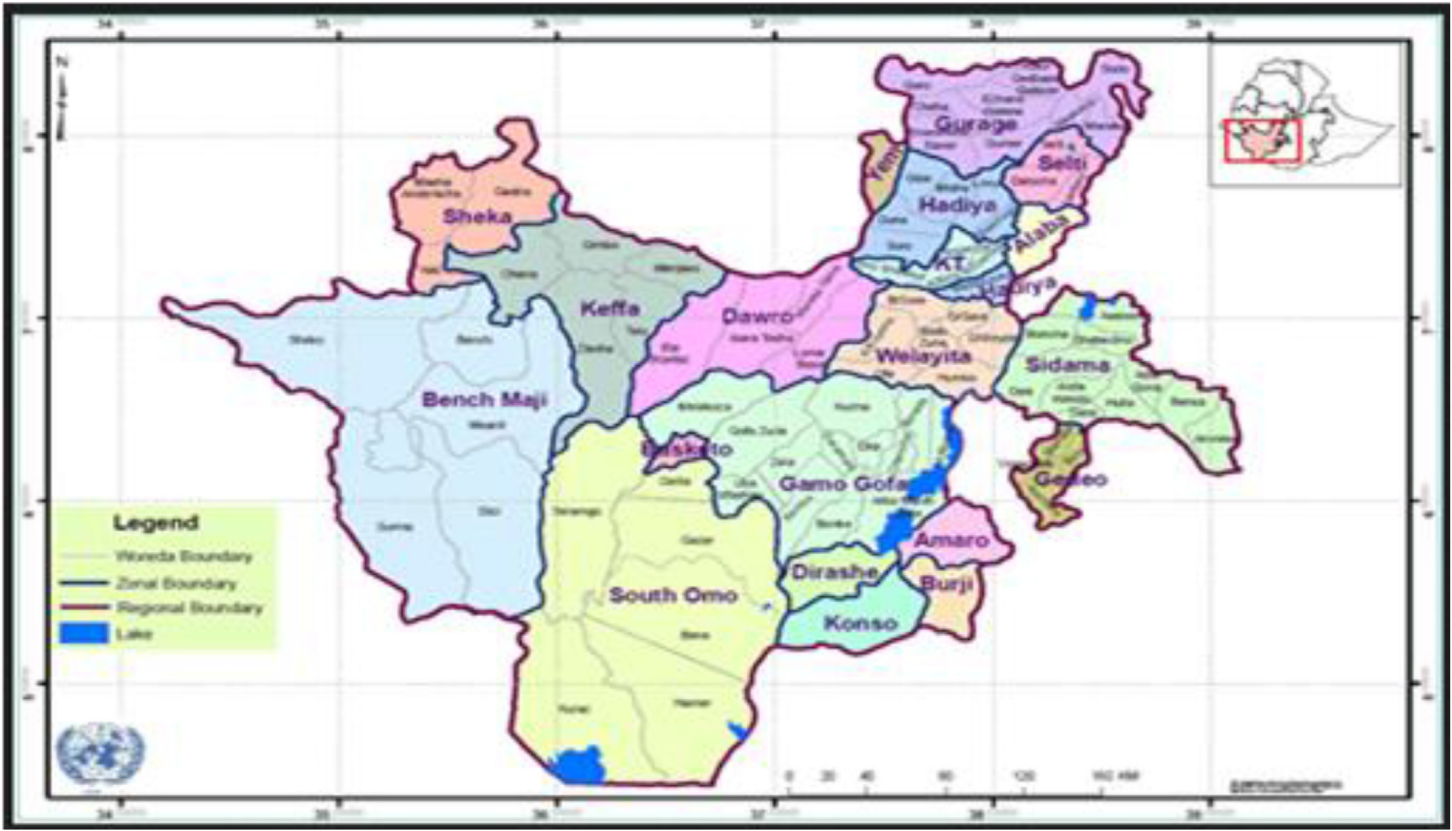
Map of south nation nationality and peoples regional state.

**Figure 2:**
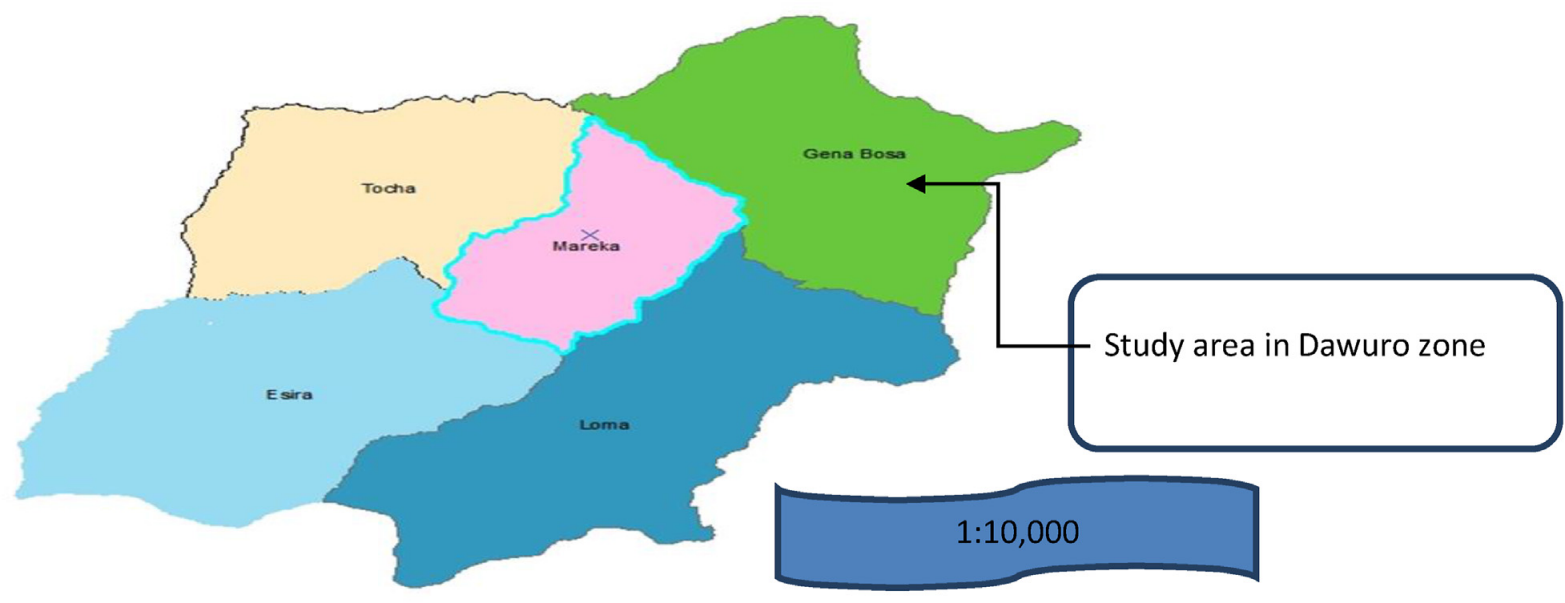
Map of Dawro zone and study district.

The research was carried out in the Gena Bossa district (Figure 2). The district is located in the South Nation Nationalities and Peoples Region State's Dawro zone (SNNPRS) (Figure 1). Karawo is the district's capital, located 508 km southwest of Addis Ababa, across Shashemene and Wolayita, and 303 km away from the SNNPRS town of Hawassa. The district occupies a land area of 90,122 ha. The Gena Bossa district has an overall population of 109,401 people, with 54,870 male and 54,531 female. In the study area, there are 19, 159 housing units. Gena Bossa district is classified into three agro-ecological zones, namely lowland (kola), midland (Woyna Dega), and highland (Dega) which covers 50%, 38.8%, and 11.2%, respectively. The annual mean temperature ranges from 16.1 to 28oc. Temperature ranges 16.1–20, 21–24.1 and 24.2–28^**o**^C in highland, midland and lowland agro-ecologies, respectively. The rainfall is a bimodal type: the short rainy season is between (February to March) and the long rainy season is between (May to September). The average annual rainfall ranges from 500mm to 1,200mm (Matawork et al., 2018). The rainfall ranges 901–1200, 701–900 and 500–700mm in highland, midland and lowland agro-ecologies, respectively (Gena Bossa Woreda Agricultural Office, 2020).

According to the land utilization data of the district; 34.8% is cultivated land, 17.5% is grazing land, 27.7% is forested, bushes, and shrubland, and 20% is covered by others. The total livestock of the districts were 287046, 77350, 84750, 277, 4440, 4000 and 147780 cattle, sheep, goats, horses, mules, donkeys and chickens, respectively. Crop production was also the main source of economy and highly cultivated crops were maize, tiff, inset, cotton, peas, beans, and spices.

Source:-By Ted Staffer of De Birhan Media 27/10/2011.

### 2.2 Data collection methods

#### 2.2.1 Evaluation of egg quality

The study area was clustered to three agro-ecologies, namely; Highland, Midland and Lowland. Then households those live in those agro-ecology also divided in to four education categories, namely; illiterate, reading and writing, primary first cycle and primary second cycle. Finally, from each agro-ecology 12 households were selected (highland, midland and lowland agro-ecologies). Also from each education level 9 households were selected (illiterate, reading and writing, primary first cycle and primary second cycle) (Table-1).

**Table-1:** Sampling frame of households in the study area.

Agro-ecology	Number of households on each agro-ecology	Number of households on each education level			
		Illiterate	R&W	PFC	PSC
Highland	12	3	3	3	3
Midland	12	3	3	3	3
Lowland	12	3	3	3	3
Total	36	9	9	9	3

Eggs laid during seven days were collected from 12 households from each agro-ecologies and a total of 36 households were randomly selected for collections of fresh eggs. Those households were based on education level and nine households were selected from each education level (illiterate, reading and writing, primary first cycle, and second cycle) of farmers. A total of 108 eggs were required for the experiment, three eggs were collected from each household, 36 eggs from each agro-ecologies, and 27 eggs from each education level of farmers collected by getting especial agreement with producers to purchase fresh eggs (Table-1). Eggs were transported on the eighth day of laying and the qualities of the egg were measured on the ninth day. After purchasing eggs, eggs were transported to the Jimma University College of Agriculture and Veterinary Medicine (JUCAVM) poultry laboratory for quality analysis. The eggs were coded at purchasing time according to the agro-ecology and education level of producers. Both internal and external egg quality parameters were measured for all eggs purchased for this aim.

Egg qualities were measured in terms of egg weight, shell thickness, albumen weight, albumen height, yolk weight, yolk height, yolk color, shell weight, and egg breaking strength. Each egg weight was measured by employing a two-digit touchy electrical adjustment and carefully broken onto a level surface. The yolk and albumen were carefully isolated. The yolk weight and fresh shell weight were measured by utilizing the same two-digit touchy electrical adjustment. The weight of albumen was calculated by subtracting the weight of the yolk and shell from the total weight of the egg. The thick albumen height (AH) was measured at its most extensive portion at a position midway between the yolk and the outer edge by employing a tripod micrometer gauge. Yolk height was moreover measured by employing a tripod micrometer gage. Eggshell thickness was measured at the center, wide estimate, and limit estimate of the shell by utilizing calibrated micrometer screw gauge, and the normal esteem was taken. Eggshell breaking strength was measured by an egg force reader manufactured by Orka food technology. Yolk color was measured using roach color fun measurement strips which range 1–15 strips from pale to orange-yellow. Haugh unit measures the egg quality and it was calculated by using the following formula adopted from (Haugh, 1937).

HU=100log(AH+7.57–1.7EW0.37)

Where: HU = Haugh unit,

AH = albumen height in mm and.

EW = egg weight in grams.

#### 2.2.2 Data management and analysis

The numerical data were calculated to the analysis of variance (ANOVA) using the general linear model procedure of SPSS. ANOVA model statement was used to examine the consequences of altitude difference and education level of farmers on the internal and external egg quality parameters were analyzed by a general linear model using SPSS.

### Statistical model for this study

Model for evaluation of egg quality test (experiment) is shown below.

Yijk = μ + Ai + Ej + (AE)ij + Єijk.

Where Y_ijk_ = the value of respective variable mentioned above about the ith agro-ecology (i = 3, highland, midland or lowland), jth education level and ijth interaction effect on egg quality.

μ = overall mean of the respective variable.

Ai = the effect of i^th^ agro-ecology (i = 3; highland, midland, or lowland) on the respective variable on egg quality.

Ej = the effect of the j^th^ educational level of producer.

(AE)ij = the interaction effect of i^th^ agro-ecology and j^th^ educational level.

Єijk = random error term.

If there is a significant difference between treatments, the Least Significant Difference (LSD) test was used to determine mean separation.

## 3 Results and discussions

### 3.1 Egg quality parameters

#### 3.1.1 External quality of eggs

In this study, external qualities of eggs were measured in terms of egg weight, fresh shell weight, shell thickness, and eggshell breaking strength. Based on this study, the average weight of eggs was 42.41 ± 0.45g (Table 2). According to UNECE (2010), a commercial egg quality standard egg with an average weight of less than 53g is a small egg. Based on this standard, the present result lies on small egg categories. Similar egg weight with present finding was reported by Bikila et al. (2016) and Fereja et al. (2016) from Chelliya district of Western Shewa in which egg weight of village chicken was 41.13g. The result of the present study on the egg weight (Table 2) for indigenous chicken reared in Dawuro Zone Gena Bossa district was similar with the finding of several researchers from Ethiopia (Kejela et al., 2019; Emebet, 2015; Mesere, 2010; Aberra et al., 2012). Comparably highest egg weight was reported from the Gorogutu district from village chickens that uses a scavenging system of production which was 45.75g (Ahmedin and Mangistu, 2016). In this study, the average egg shell thickness was 0.51 ± 0.07mm. Halima (2007) reported the thickest shell thickness (0.71mm) from intensively managed local chicken ecotypes in North West Amhara. In current investigation, 5.04 ± 0.39g was the average eggshell weight (Table 2). Comparably similar shell weight was reported from Gorogota district from village chickens which were 4.95g (Ahmedin and Mangistu, 2016). The average eggshell breaking strength in this study was 3.63 ± 0.11kgf. Eggshell strength is important for chicken producers because lower strength causes a higher percentage of broken eggs increasing the economic losses (Ketta and Tumova, 2016). Zita et al. (2009) observed that eggshell strength was higher from the onset of lay till the end of the first phase and declined afterward. However, Pavlik et al. (2009) found that the eggshell breaking strength of birds declines with age. Mertens et al. (2006) investigated the effects of various housing systems (conventional cages, enriched cages, aviary, and free-range) on eggshell quality, finding that free-range chicken eggs had the weakest shell strength. Eggshell strength was highly related to the chicken management system especially feeding (minerals like Calcium and phosphorus) (Ketta and Tumova, 2016).

**Table-2:** The effect of agro-ecology and education level of farmers on egg quality (Mean +SE).

Parameters	Agro-ecology				Education level					A∗E	Overall
	HL	ML	LL	p-value	Illi.	R&W	PFC	PSC	p-value		
						
**EW (g)**	43.94 ±0.42^**a**^	42.59 ± 0.42^**a**^	40.71 ± 0.42^**b**^	0.001	40.80 ± 0.48^**b**^	41.02 ±0.48^**b**^	43.42 ± 0.48^**a**^	44.41 ± 0.48^**a**^	0.001	∗∗	42.41 ± 0.45
**ST (mm)**	0.54± 0.01^**a**^	0.51 ± 0.01^**b**^	0.49 ± 0.01^**b**^	0.001	0.54 ± 0.11^**a**^	0.48± 0.11^**b**^	0.52 ± 0.11^**ab**^	0.50 ± 0.11^**ab**^	0.005	∗∗	0.51 ± 0.07
**SW (g)**	5.25± 0.73^**a**^	5.12 ± 0.73^**a**^	4.76 ± 0.73^**b**^	0.001	4.85 ± 0.08^**b**^	4.86± 0.08^**b**^	5.13 ± 0.08^**ab**^	5.33 ± 0.08^**a**^	0.001	NS	5.04 ± 0.39
**ES (kgf)**	3.71± 0.11	3.66 ± 0.11	3.52 ± 0.11	0.389	3.64 ± 0.12	3.55± 0.12	3.72 ± 0.12	3.61 ± 0.12	0.803	NS	3.63 ± 0.11
**HUS (%)**	69.06 ± 1.22	66.37 ± 1.22	70.92 ± 1.22	0.412	67.71 ± 1.41^**b**^	63.86 ± 1.41^**b**^	68.24 ± 1.41^**b**^	75.34 ± 1.41^**a**^	0.001	NS	68.78 ± 1.33
**YH (mm)**	15.37 ± 0.28^**a**^	15.06 ± 0.28^**a**^	14.08 ± 0.28^**b**^	0.001	13.77 ± 0.33^**b**^	14.81 ± 0.33^**ab**^	15.18 ± 0.33^**a**^	15.58 ± 0.33^**a**^	0.001	NS	14.83 ± 0.31
**AW (g)**	23.31 ± 0.40^**a**^	21.77 ± 0.40^**b**^	21.27 ± 0.40^**b**^	0.001	20.61 ± 0.46^**c**^	21.37 ± 0.46^**bc**^	22.84 ± 0.46^**b**^	23.65 ± 0.46^**a**^	0.001	∗∗	22.12 ± 0.43
**YC**	8.15± 0.14^**b**^	8.33 ± 0.14^**b**^	8.65 ± 0.14^**a**^	0.01	8.42 ± 0.16	8.36± 0.16	8.28 ± 0.16	8.44 ± 0.16	0.181	NS	8.37 ± 0.15
**YW (g)**	15.37 ± 0.25^**ab**^	15.69 ± 0.25^**a**^	14.58 ± 0.25^**b**^	0.01	15.27 ± 0.29	14.74 ± 0.29	15.48 ± 0.29	15.36 ± 0.29	0.285	NS	15.21 ± 0.27
**AH (mm)**	4.29± 0.15	3.94 ± 0.15	4.41 ± 0.15	0.172	3.96 ± 0.17^**b**^	3.56± 0.17^**b**^	4.16 ± 0.17^**b**^	5.17 ± 0.17^**a**^	0.001	NS	4.21 ± 0.16

There were significant (p < 0.05) differences in egg weight, shell thickness, and shell weight at different agro-ecologies and education levels but there was no significant (p > 0.05) difference in egg strength at different agro-ecologies and education levels in this study. Based on this study, the egg weight of lowland (40.71 ± 0.42g) was significantly (p < 0.001) lowest than midland (42.59 ± 0.42g) and highland (43.94 ± 0.43g) eggs (Table 2). There could be a reason for the difference such as the extreme temperature of the lowland affects egg weight by evaporating water through the pores of the eggshell. It could also be due to poor egg handling and storage conditions prior to collection. This result was in agreement with Samli et al. (2005), who reported that HU and egg weight is highly affected by egg storage time and storing temperature. The highest egg weight was observed from primary first (43.42 ± 0.48g) and primary second (44.41 ± 0.48g) cycle-educated farmers (Table 2). This difference may be observed due to good management practice given from PFC and PSC educated respondents in terms of feeding, health managements and providing comfort environment for chickens. However, the main factors that affecting internal and external quality of the eggs requires further investigations in study area.

Shell thickness of the eggs in this study was 0.54 ± 0.01, 0.51 ± 0.01, and 0.49 ± 0.01 mm at highland, midland, and lowland areas, respectively (Table 2). According to Yamak et al. (2015) classification eggs with shell thickness less than 0.35mm is thin, between 0.35-0.38mm is medium, and greater than 0.38mm is thick eggshell. Eggshell strength was stronger in the eggs of thick shell category (Mohamed and Eva, 2017). Aberra et al. (2013) found that eggs collected from scavenging village chickens reared in different ago-ecological zones of Ethiopia had the thinnest eggshells, measuring 0.296mm. The lowest eggshell thickness was obtained from lowland and the highest eggshell thickness was obtained from highland agro-ecologies. This difference could be due to changes through environmental conditions and the type of chicken feed provided by farmers. It agrees with Fanu et al. (2019), the difference of shell thickness of Sasso T44 chicken in North Showa Zone is agro-ecological variation as well as feed type which affects egg shell thickness; because as the temperature level increased the chicken feed intake decreased, the consequence not getting enough calcium for egg shell formation. This result agrees with Zita et al. (2009) report the effect of layer type difference, environmental conditions and feed quality affects shell thickness. Eggs collected from illiterate farmers had the thickest shell (0.54 ± 0.11mm) than reading and writing (0.48 ± 0.11mm), primary first (0.52 ± 0.11), and second cycle (0.50 ± 0.11) educated farmers (Table 2). The lowest shell thickness was measured from the reading and writing education levels of the farmers. This is because illiterate farmers feed their chickens different types of feed at different times of the day, resulting in thicker eggshells than other farmers.

Shell weight in this study was 5.25 ± 0.73, 5.12 ± 0.73, and 4.76 ± 0.73 g at highland, midland, and lowland agro-ecologies, respectively (Table-2). The lowest shell weight was measured from lowland agro-ecologies and it might be eggs affected by the highest temperature of the agro-ecology before collection. The average shell weight in the Gorogutu district was 4.7g, 4.8g, and 5.3g in the highland, midland, and lowland areas, respectively (Ahimedin and Mangistu, 2016). The highest eggshell weight was measured from eggs that were collected from primary second cycle (5.33 ± 0.08g) educated farmers and the lowest eggshell weight was obtained from eggs that were collected from illiterate (4.85 ± 0.08g) and reading and writing (4.86 ± 0.08g) education level of farmers (Table 2). This difference in egg weight and shell weight might be due to educated farmers giving better management such as feeding to layers and eggs by storing in lowest temperature and handling eggs in a better way which reduces egg deterioration. According to Gary and Richard (2015) finding eggshell quality was affected by adequacy of nutrition, flock health problems, management practices, environmental conditions, and breeding.

**Figure-3:**
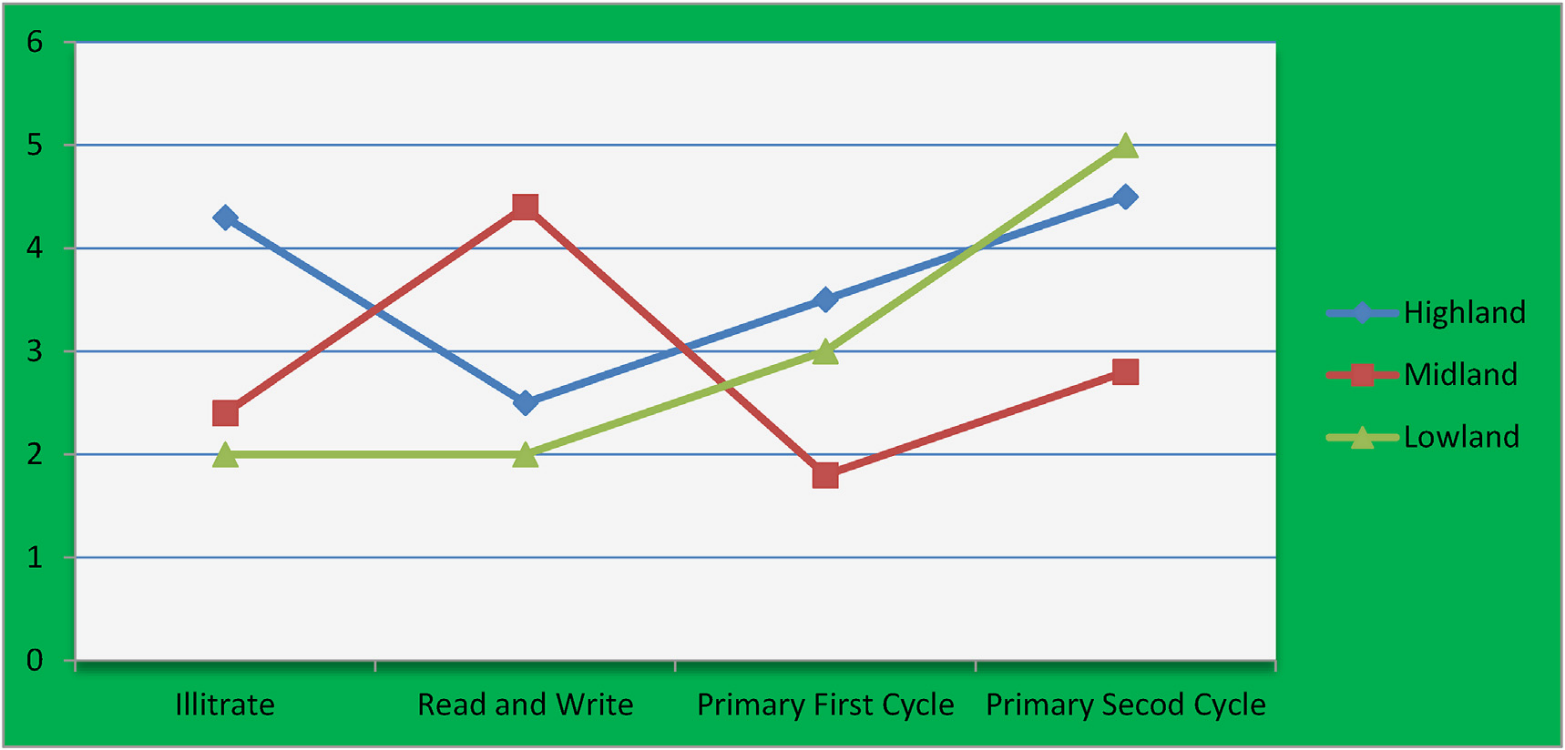
Agro-ecology and Education level interaction effect in the egg quality.

There were significant (p < 0.001) differences in egg weight and eggshell thickness at the interaction point of agro-ecology and the education level of the farmers (Figure 3). This means at proper agro-ecology the educated farmers provide better management for egg-laying chickens and after the eggs were laid educated farmers collect and store them in a proper place to reduce quality deterioration. Also, they might handle eggs in better ways and store them in the correct position (large side up position). Egg weight was most appropriate in primary first cycle and primary second cycle interactions with highland and midland agro-ecologies. However, shell strength was most appropriate in the interactions of highland agro-ecology with illiterate education level (Table 3). This difference might occurred due to in highland and midland agro-ecologies PFC and PSC educated farmers provided appropriate managements for their chickens as well as they collects and stores appropriately in good place. It might be also the environmental temperature rise was low when comparing to lowland agro-ecologies.

**Table-3:** Egg quality for interaction effect of agro-ecology and education level of farmers.

Variables	DF	Mean square	F-value	Sig.
**Egg weight (g)**	6	32.662	3.904	0.001
**Shell thickness (mm)**	6	0.015	3.198	0.006
**Albumen height (mm)**	6	1.051	1.006	0.424
**Yolk color**	6	1.118	1.247	0.286
**HUS (%)**	6	71.200	0.994	0.432
**Yolk height (mm)**	6	2.787	0.709	0.643
**Albumen weight (g)**	6	17.557	2.265	0.041
**Shell weight (g)**	6	0.412	1.616	0.147
**Yolk weight (g)**	6	4.567	1.539	0.170
**Egg strength (kgf)**	6	0.294	0.560	0.762

#### 3.1.2 Internal qualities of eggs

Internal qualities of eggs were measured in terms of albumen height, albumen weight, yolk height, yolk weight, and yolk color. Also, HUS is calculated from egg weight and albumen height. Albumen height in this study was 4.21 ± 0.16mm (Table 2). This result agreed with 4.37mm reported by Fereja et al. (2016) for village chickens in the Chelliya district of Western Shoa. In this study, the mean albumen weight and yolk height were 22.12 ± 0.43g and 14.83 ± 0.31mm, respectively. This result agrees with Fisseha et al. (2010) report in which yolk height was 15.1mm for eggs collected from Bure, Fogera, and Dale districts. The yolk weight of the egg collected from the different areas was 15.21± 0.27g. The lowest and highest yolk weight was reported by Fisseha et al. (2010) from village chickens of Bure and Fogera district that were 14.6g and 16.28g, respectively. The average yolk color of eggs in this study was 8.37 ± 0.15. This result was similar to Fisseha et al. (2010) result in which village chicken eggs' yolk color was 8.6 for eggs collected from Bure, Fogera, and Dale districts. The average Haugh unit score in the present study was 68.78 ± 1.33% (Table 2). This result was in agreement with Bikile (2013) and Fereja et al. (2016) report in which HUS for indigenous chicken eggs collected from the Chelliya district was 69.13%. Halima (2007) reported the lowest HUS value (61.1%) from eggs collected from local ecotypes in North-West Amhara. Highest HUS was reported by Ahmedin and Mangistu (2016) in the Gorogutu district from rural chickens which were 75.69%. The Haugh unit is mainly influenced by the albumen height and egg weight (Serkalem et al., 2019).

There were significant (p < 0.05) differences in albumen weight, yolk height, yolk weight, and yolk color at different agro-ecologies, respectively (Table 2). On the opposite of this, there were no significant (p > 0.05) differences in albumen height and HUS in this study at different agro-ecologies. Eggs collected from highland (23.31 + 0.40g) had the highest albumen weight than eggs collected from lowland (21.27 + 0.40g) and midland (21.77 + 0.40g) agro-ecologies. This result agrees with Ahimedin and Mangistu's (2016) report in which the mean albumen weight was 26g, 24.9g, and 24.2 g at highland, midland, and lowland areas of Gorogutu district, respectively. The lowest yolk height was obtained from lowland (14.08 ± 0.28mm) and the highest yolk height was obtained from highland (15.37 ± 0.28mm) and midland (15.06 ± 0.28). Aberra et al. (2013) found that the yolk height of indigenous chickens reared in Ethiopia's highland, midland, and lowland altitudes was 16.1mm, 16.4mm, and 16.0mm, respectively. Lowland eggs had significantly highest yolk color (8.65 ± 0.14) than highland (8.15 ± 0.14) and midland (8.33 ± 0.14) eggs (Table 2). Comparably highest yolk color was reported by Bikila et al. (2016) from the Chelliya district of western Shewa the average yolk color was 10.20 and 9.58 at highland and midland agro-ecologies, respectively. In this study, lowland scavenging feed resources might contain the highest amount of xanthophyll than highland scavenging feed resources, which affects yolk color. The lower yolk colour is due to chicken feed household leftover feed type, whereas high yolk colour (deep in yellowish colour) is due to the chicken are feed green plant and grain feed (Fanu et al., 2019). The amount of xanthophyll (plant pigment) in the diet determines the colour of the yolk (Silversides et al., 2006). Carotenoid deposits in the yolk, which improve yolk colour, may be caused by scavenging on green grass. On the other hand, only supplementing maize is used to improve the yolk color of the egg. The colour of the eggs yolk is determined by the feed (Solomon, 2004). However, genetic factors influence the variation of yolk color under the controlled feeding environments (Emília et al., 2015). Getachew (2019) also reported that the yolk color score of free range local hens was higher compared to eggs collected from hens managed under intensive chicken management condition. When it comes to egg quality, the colour of the yolk is a critical consideration for any consumer (Okeudo et al., 2003). Consumer preferences for yolk colour are extremely subjective and vary greatly by country. According to Aberra et al. (2013) and Okeud et al. (2003) reports, yolk color is a key factor in any consumer survey relating to egg quality, consumer preferences for yolk color are highly subjective and vary widely from country to country. Ethiopian consumers prefer the deep yellow colour of eggs from small scavenging local chickens over the pale yolk colour of larger eggs from improved strains (Tadelle et al., 2003).

There were significant (p < 0.001) differences in albumen height, albumen weight, yolk height, and HUS for eggs collected from different education levels of farmers. But yolk weight and yolk color were not significantly (p > 0.05) different for eggs collected from different education levels of farmers (Table-2). The highest albumen height was obtained from primary second cycle (5.17 ± 0.17mm) educated farmers. Highest and lowest albumen weight obtained from eggs collected from primary second cycle (23.65 ± 0.0.46g) educated and illiterate (20.61 ± 0.46g) farmers, respectively. Also, the highest yolk height obtained from primary first (15.18 ± 0.33mm) and primary second (15.58 ± 0.33mm) cycle educated farmers and lowest yolk height obtained from eggs collected from illiterate (13.77 ± 0.33mm) farmers. There was significantly (p < 0.001) highest HUS calculated from eggs collected from a primary second (75.34 ± 1.41%) cycle-educated farmers (Table 2). According to TAS (2010) report the USDA egg quality standard shows eggs with HUS greater than or equal to 73% is grade 'AA' eggs, eggs with HUS between 60-71% is grade 'A' eggs, and HUS less than 60% is grade 'B' eggs. So, eggs collected from the primary second cycle are 'AA' grade and others lie in 'A' grade. Haugh unit score is used as an indicator of egg freshness (Gary and Richard, 2015). This difference in albumen height, albumen weight, yolk height, and HUS in this result might be primary second cycle educated farmers gave care for eggs by storing in low temperature until collecting for experiment and farmers handle eggs properly to reduce egg quality deteriorations. Most of the changes in egg quality in terms of albumen height and HUS, can be attributed to water loss through evaporation through the pores in the shell and carbon dioxide escape from albumen, resulting in a progressive loss in egg weight and a continuous decline in egg quality (Samli et al., 2005).

There were significant (p < 0.001) differences in albumen weight at the interaction point of agro-ecology and the education level of the farmers (Figure-3; Table 3). This means at proper agro-ecology the educated farmers provide better management for eggs by storing proper temperature and correct position to reduce quality deteriorations. The combinations of primary second cycle with highland agro-ecology was most appropriate than other interactions for albumen weight.

## 4 Conclusion and recommendation

In the current study, highland eggs had the highest egg weight (43.94 ± 0.42g), shell thickness (0.54 ± 0.01mm), albumen weight (23.31 ± 0.40g), and shell weight (5.25 ± 0.73g). Lowland eggs had the highest yolk color score (8.65 ± 0.14). Comparing to education level, eggs collected from primary second cycle educated farmers had the highest egg weight (44.41 ± 0.48g), albumen height (5.17 ± 0.17mm), Haugh unit score (75.34 ± 1.41%), yolk height (15.58 ± 0.33), albumen weight (23.65 ± 0.46g), and shell weight (5.33 ± 0.08g). Except for shell thickness (0.54 ± 0.11mm) all egg quality parameters were lowest for eggs collected from illiterate farmers. Egg weight was most appropriate in primary first cycle and primary second cycle interactions points with respective highland and midland agro-ecologies. However, shell strength was most appropriate in the interactions of highland agro-ecology with illiterate education level. The combinations of primary second cycle with highland agro-ecology was most appropriate than other interactions for albumen weight. Depending on this conclusion, it will better to investigate further studies on factors affecting on internal and external qualities of the eggs in the study area.

• PFC = primary first cycle (grade 1–4), PSC = primary second cycle (grade 5–8), R&W = reading and writing
• ^a,ab,b^ Means within a row under the same heading with different superscript differ significantly between the agro ecologies and education level of farmers (P < 0.05); EW = Egg Weight; ST = Shell Thickness; AH = Albumen Height; YC = Yolk Color; HUS = Hough unit Score; YH = Yolk Height; AW = Albumen Weight, SW = Shell Weight; YW = Yolk Weight; ES = Egg shell breaking Strength, NS = Not Significant, SE = Standard Error, g = gram, mm = millimeter; % = percent, HL = highland, ML = midland, LL = lowland, PFC = primary first cycle, PSC = primary second cycle, R&W = reading and writing, Illi. = illiterate, kgf = kilogram force, A∗E = interaction of agro-ecology and education level, ∗∗ = Significant

## Declarations

### Author contribution statement

Matawork Milkias: Conceived and designed the experiments; Performed the experiments; Analyzed and interpreted the data; Wrote the paper.

Meseret Molla: Contributed reagents, materials, analysis tools or data.

### Funding statement

This research did not receive any specific grant from funding agencies in the public, commercial, or not-for-profit sectors.

### Data availability statement

Data will be made available on request.

### Declaration of interests statement

The authors declare no conflict of interest.

### Additional information

No additional information is available for this paper.
